# Exosomal miR-99a-5p is elevated in sera of ovarian cancer patients and promotes cancer cell invasion by increasing fibronectin and vitronectin expression in neighboring peritoneal mesothelial cells

**DOI:** 10.1186/s12885-018-4974-5

**Published:** 2018-11-05

**Authors:** Akihiko Yoshimura, Kenjiro Sawada, Koji Nakamura, Yasuto Kinose, Erika Nakatsuka, Masaki Kobayashi, Mayuko Miyamoto, Kyoso Ishida, Yuri Matsumoto, Michiko Kodama, Kae Hashimoto, Seiji Mabuchi, Tadashi Kimura

**Affiliations:** 1 Departments of Obstetrics and Gynecology, Osaka University Graduate School of Medicine, 2-2 Yamadaoka, Suita, Osaka, 565-0871 Japan; 2 Department of Molecular Oncology, H. Lee Moffitt Cancer Center & Research Institute, Tampa, FL USA; 3 Penn Ovarian Cancer Research Center, Department of Obstetrics and Gynecology, University of Pennsylvania Perelman School of Medicine, Philadelphia, PA USA

**Keywords:** Ovarian cancer, microRNA, Exosome, Biomarker, Fibronectin, Vitronectin

## Abstract

**Background:**

microRNAs (miRNAs) stably exist in circulating blood and are encapsulated in extracellular vesicles such as exosomes. The aims of this study were to identify which exosomal miRNAs are highly produced from epithelial ovarian cancer (EOC) cells, to analyze whether serum miRNA can be used to discriminate patients with EOC from healthy volunteers, and to investigate the functional role of exosomal miRNAs in ovarian cancer progression.

**Methods:**

Exosomes were collected from the culture media of serous ovarian cancer cell lines, namely TYK-nu and HeyA8 cells. An exosomal miRNA microarray revealed that several miRNAs including miR-99a-5p were specifically elevated in EOC-derived exosomes. Expression levels of serum miR-99a-5p in 62 patients with EOC, 26 patients with benign ovarian tumors, and 20 healthy volunteers were determined by miRNA quantitative reverse transcription-polymerase chain reaction. To investigate the role of exosomal miR-99a-5p in peritoneal dissemination, neighboring human peritoneal mesothelial cells (HPMCs) were treated with EOC-derived exosomes and then expression levels of miR-99a-5p were examined. Furthermore, mimics of miR-99a-5p were transfected into HPMCs and the effect of miR-99a-5p on cancer invasion was analyzed using a 3D culture model. Proteomic analysis with the tandem mass tag method was performed on HPMCs transfected with miR-99a-5p and then potential target genes of miR-99a-5p were examined.

**Results:**

The serum miR-99a-5p levels were significantly increased in patients with EOC, compared with those in benign tumor patients and healthy volunteers (1.7-fold and 2.8-fold, respectively). A receiver operating characteristic curve analysis showed with a cut-off of 1.41 showed sensitivity and specificity of 0.85 and 0.75, respectively, for detecting EOC (area under the curve = 0.88). Serum miR-99a-5p expression levels were significantly decreased after EOC surgeries (1.8 to 1.3, *p* = 0.002), indicating that miR-99a-5p reflects tumor burden. Treatment with EOC-derived exosomes significantly increased miR-99a-5p expression in HPMCs. HPMCs transfected with miR-99a-5p promoted ovarian cancer invasion and exhibited increased expression levels of fibronectin and vitronectin.

**Conclusions:**

Serum miR-99a-5p is significantly elevated in ovarian cancer patients. Exosomal miR-99a-5p from EOC cells promotes cell invasion by affecting HPMCs through fibronectin and vitronectin upregulation and may serve as a target for inhibiting ovarian cancer progression.

**Electronic supplementary material:**

The online version of this article (10.1186/s12885-018-4974-5) contains supplementary material, which is available to authorized users.

## Background

Ovarian cancer, which has a survival rate of 30%, is generally characterized by widespread peritoneal dissemination and ascites and is the leading cause of death among gynecological cancers [1]. In the United States, ovarian cancer is the fifth leading cause of cancer death in women and is responsible for an estimated 14,080 deaths annually, with 22,400 patients diagnosed in 2017 [2]. The dismal outcomes of ovarian cancer are mainly due to late-stage diagnosis as it is usually asymptomatic until later stages. In patients with stage III or IV cancer, as determined by the International Federation of Gynecology and Obstetrics (FIGO) staging system, the 5-year survival rate remains at less than 30% and has not improved since the late 1990s despite comprehensive treatment with aggressive cytoreductive surgery and chemotherapy using platinum and taxane-based drugs. On the other hand, the few patients diagnosed at stage I have a 5-year survival rate of over 90% [3], indicating that the early detection of ovarian cancer can directly and drastically improve patient prognoses. While pelvic examination, transvaginal ultrasonography, and serum carbohydrate antigen 125 (CA125) tests are performed during routine diagnostic procedures, they typically fail to detect the disease at an early stage and thus have not reduced the mortality rate of ovarian cancer significantly [4]. Indeed, CA125 is only elevated in 50–60% of patients with stage I-II ovarian cancer [5]. Therefore, there is a critical need for improved diagnostic markers to detect ovarian cancer.

MicroRNAs (miRNAs) are small (19–25 nucleotide), non-coding, RNA molecules that play various roles in physiology and disease development. miRNA dysfunctions have been extensively reported in various cancers including ovarian cancer. Furthermore, emerging evidence has shown that miRNAs exist not only in cells but also in circulating blood, reflecting tissue or organ conditions. Circulatory miRNAs in blood are resistant to the degradation of RNase enzymes and remain stable [6], indicating that they can potentially serve as novel diagnostic or prognostic markers. One major reason why miRNA remains undegraded in circulation is that miRNAs are encapsulated in extracellular membrane vesicles such as exosomes [7]. Exosomes are small membrane vesicles that are approximately 100 nm in diameter and contains proteins, lipids, mRNA, and miRNA. Tumor-specific exosomes are shed by tumor cells and enter the circulation; therefore, exosomal miRNAs in body fluids may be useful diagnostic biomarkers for the detection of cancer [8]. However, robust studies evaluating circulatory exosomal miRNA signatures in ovarian cancer have yet to be reported and there is a lack of information regarding the relationship between exosomal miRNA profiles in circulation and the pathological condition of ovarian cancer patients.

With these facts in mind, we collected exosomes from the culture media of serous epithelial ovarian cancer (EOC) cell lines, performed miRNA microarrays, and found that miR-99a-5p is specifically elevated in EOC-derived exosomes. To evaluate the potential of miR-99a-5p as a cancer biomarker, the expression levels of serum miR-99a-5p in EOC patients were analyzed and compared with those of patients with benign ovarian tumors and healthy controls. Furthermore, we investigated the role of exosomal miR-99a-5p during ovarian cancer progression, focusing on exosomal transfer to human peritoneal mesothelial cells (HPMCs) as ovarian cancer cells attach to HPMCs covering the peritoneum, omentum, or bowel serosa as the initial step of peritoneal dissemination.

## Methods

### Materials

Dulbecco’s modified Eagle’s medium (DMEM) and Roswell Park Memorial Institute (RPMI) 1640 medium were obtained from Nacalai Tesque (Kyoto, Japan). Fetal bovine serum (FBS; #172012) was purchased from Sigma Aldrich (St. Louis, MO). Antibody against CD63 (#11–343-C025) was purchased from EXBIO (Prague, Czech Republic). Donkey anti-mouse IgG 10 nm gold (#ab39593) was purchased from Abcam (Cambridge, UK). Growth factor-reduced basement membrane protein (Matrigel; #356230) was purchased from Corning (New York, NY). Antibodies against vitronectin 65/75 (D-8; #sc-74,484) and fibronectin (EP5; #sc-8422) were purchased from Santa Cruz Biotechnology (Dallas, TX). Antibody against β-actin (#4967) was purchased from Cell Signaling Technology (Danvers, MA).

### Cell culture

The HeyA8 cell line was generously provided by Dr. Anil Sood (MD Anderson Cancer Center, Houston, TX) in 2010 and the TYK-nu cell line was purchased from Health Science Research Resources Bank (JCRB0234.0; Osaka, Japan) in 2011. Cells were authenticated by short tandem repeat DNA profiling at Takara-Bio Inc. (Otsu, Japan) and were used for this study within 6 months of resuscitation. The IOSE cell line was generously provided by Dr. Masaki Mandai (Kyoto University, Kyoto, Japan) in 2013. Briefly, ovarian surface epithelial cells were collected from normal ovaries and transfected with SV40 large T antigen and human telomerase reverse transcriptase [9]. HPMCs were isolated from the omentum of patients undergoing surgery at Osaka University Hospital as previously described [10]. Written informed consent was obtained from each patient before surgery. Briefly, small pieces of omentum were trypsinized at 37 °C for 30 min and the cell-containing suspension was filtered through a 200-nm pore nylon mesh and centrifuged at 500×*g* for 5 min. The cells were cultured in RPMI 1640 supplemented with 20% FBS, 100 U/mL penicillin, and 100 μg/mL streptomycin and incubated at 5% CO_2_ and saturated humidity at 37 °C. The cells were harvested during the second or third passage after primary culture for experiments. Mycoplasma contamination had been routinely checked using EZ-PCR Mycoplasma Test Kit (Biological Industries, Kibbutz Beit Haemek, Israel).

### Exosome preparation

Conditioned medium (CM) containing exosome-depleted FBS (prepared by overnight ultracentrifugation at 100,000×*g* at 4 °C) was prepared by incubating cells grown at subconfluence for 48 h. CM was centrifuged at 2000×*g* for 10 min at 4 °C and the supernatant fraction was filtered through 200-nm pore size filters. The resulting cell-free medium was ultracentrifuged at 100,000×*g* for 70 min at 4 °C using a Beckman™ L-90 K ultracentrifuge (Brea, CA). The supernatant fraction was discarded, and then the exosome-containing pellet was resuspended in phosphate-buffered saline (PBS) and ultracentrifuged under the same conditions. The pellet was finally resuspended in PBS and the amount of exosomal protein was assessed by the Lowry method (Bio-Rad, Hercules, CA).

### Electron microscopy

Electron microscopy was performed as described using a transmission electron microscope (H-7650; Hitachi, Ltd., Tokyo, Japan).

### Measurement of exosome particle size distribution

Exosome suspensions were diluted 1000-fold with PBS and nanoparticle tracking analysis was carried out using a NanoSight LM10V-HS particle analyzer (Malvern Instruments Ltd., Worcestershire, UK).

### Profiling of cellular and exosomal RNA

Total RNA was extracted using TRIzol reagent (#15596–018; Life Technologies, Carlsbad, CA:). RNA isolated from cells and exosomes was analyzed using an Agilent 2100 Bioanalyzer (Agilent Technologies, Inc. Santa Clara, CA).

### Exosomal miRNA microarray

miRNA microarrays using the GeneChip miRNA 4.0 Array (Affymetrix, Santa Clara, CA) were performed and analyzed by Filgen (Nagoya, Japan). Briefly, 1000-ng miRNA samples were biotin-labeled using a Flash Tag_TM_ Biotin HSR RNA Labeling Kit for Affymetrix GeneChip miRNA arrays (Affymetrix) according to the manufacturer’s protocol. Hybridization solution was prepared using 110.5 μL hybridization master mix and 21.5 μL biotin-labeled sample. The array was incubated using the GeneChip Hybridization Oven 645 (Affymetrix) and washed using the GeneChip Fluidics Station 450 (Affymetrix) according to the manufacturer’s protocol. The washed array was analyzed using the GeneChip Scanner 3000 7G (Affymetrix).

### Quantitative reverse transcription polymerase chain reaction (qRT-PCR) of miR-99a-5p

miRNA qRT-PCR was performed using the StepOnePlus Real-Time PCR System (Applied Biosystems, Foster City, CA). Total RNA was transcribed into cDNA using the TaqMan MicroRNA Reverse Transcription Kit (#4366596; Applied Biosystems). Mature miR-99a-5p was assayed using the TaqMan assay (#A25576; hsa-miR-99a-5p). To normalize miRNA expression levels, cel-miR-39 (#4427975; Applied Biosystems) was used as an exogenous control for serum miRNA, and RNU6B (Applied Biosystems; #001093) was used as an endogenous control for cellular miRNA. Each qRT-PCR assay was performed in triplicate, and the relative expression levels of miR-99a-5p were calculated using the 2^-∆∆Ct^ method.

### Patients and samples

Blood samples were collected from healthy volunteers (*n* = 20), patients with benign ovarian tumors (*n* = 26), and ovarian cancer patients (*n* = 62) with approval from the Institutional Review Board of Osaka University. Written informed consent was obtained from every participant before sample collection. From 26 ovarian cancer patients, blood samples were collected twice; once at admission for primary debulking surgery (PDS; 2 or 3 days before surgery) and once at admission for initial postoperative chemotherapy (36 ± 12 days after surgery). Written informed consent was obtained from every participant for the use of their samples. Blood samples were centrifuged at 1500×*g* for 10 min at 4 °C and the upper sera phases were transferred to new tubes and stored at − 80 °C until further use.

### miRNA extraction

miRNA was extracted from 200-μL serum samples using the miRNeasy Serum/Plasma Kit (Qiagen, Hilden, Germany) following the manufacturer’s protocol.

### HPMC-coated in vitro invasion assay

In miRNA experiments, miRNAs were used to transfect HPMCs after reaching 60–70% confluence. After 24 h, HPMCs were harvested and plated onto 25-μg Matrigel-coated 24-well cell culture inserts with 8-μm pore membranes (#353097; Corning). After HPMCs reached confluence, 5 × 10^4^ ovarian cancer cells were plated onto the HPMC monolayer with 0.1% bovine serum albumin (BSA)/DMEM and allowed to invade for 24 h. Cell culture media consisting of 10% FBS/DMEM was placed in the lower chamber as a chemoattractant. Once the HPMCs reached confluence in exosome experiments, the culture media was changed to 0.1% BSA/DMEM with 100 μg/mL exosomes 24 h before treatment with cancer cells. Noninvading cells were removed with a cotton swab and invading cells that migrated to the trans-side of the membrane were fixed with methanol, stained with Giemsa, and counted using the image analysis application of the BZ-X700 microscope (Keyence, Osaka, Japan).

### Transfection of miRNA

HPMCs were transfected with precursor miRNA (hsa-miR-99a-5p: #AM17100) or inhibitor miRNA (anti-miR-99a-5p; #AM17000) at a concentration of 30 nM. Pre-miR miRNA precursor negative control #1 (#AM17110) was used as a control. All oligonucleotides were purchased from Thermo Fisher Scientific (Waltham, MA). Oligonucleotide transfection was performed using Lipofectamine 3000 (#L3000–008; Thermo Fisher Scientific) according to manufacturer’s instructions. One day after transfection (24 h), cells were collected for subsequent procedures.

### Proteome analysis with tandem mass tag (TMT) system

HPMCs were transfected with miR-99a-5p or negative control miRNA for 24 h and lysed with a buffer composed of 50 mM Tris-HCl pH 7.5, 2% sodium deoxycholate, and protease inhibitor (Nacalai Tesque). Protein extracts were reduced, alkylated, and digested overnight. Samples were labeled with TMT 6-plex reagents (Thermo Fisher Scientific) and then mixed before sample fractionation and clean-up. Labeled samples were analyzed by liquid chromatography coupled with tandem mass spectrometry (LC-MS/MS) using a Q Exactive Mass Spectrometer (Thermo Fisher Scientific) equipped with an UltiMate 3000 Nano LC System (Thermo Fisher Scientific). Raw data were processed by Mascot v2.1 (Matrix Science, London, UK). Protein expression levels were compared between the two samples and pathway annotation for differentially expressed proteins was performed using DAVID 6.8 analysis (https://david.ncifcrf.gov).

### Western blot analysis

A total of 5 × 10^5^ cells were plated onto 6-well plates and lysed with radioimmunoprecipitation assay buffer (RIPA buffer; 25 mM Tris-HCl pH 7.6, 150 mM NaCl, 1% sodium deoxycholate, 1% NP-40, and 0.1% sodium dodecyl sulfate). Lysates were separated by 5–20% sodium dodecyl sulfate-polyacrylamide gel electrophoresis and transferred to polyvinylidene difluoride membranes, followed by incubation with primary antibodies (vitronectin, 1:1000 in 5% BSA; fibronectin, 1:1000 in 5% BSA; β-actin, 1:2000 in 5% BSA), and then incubation with the corresponding secondary horseradish peroxidase–conjugated IgG. The proteins were visualized with an electrochemiluminescent system (PerkinElmer Life Science, Waltham, MA).

### Statistical analysis

Statistical analysis was performed using R version 3.2.3 and JMP version 13.0.0 (SAS Institute Japan Ltd., Tokyo, Japan). Unless otherwise stated, the data are presented as the mean ± standard deviation (SD), and statistical significance was determined by a Student’s *t*-test. Multiple comparison analysis was performed by analysis of variance followed by Holm’s step-down method. In graphs showing relative expression levels of miR-99a-5p in patient sera, the box-and-whisker plots indicate median, interquartile range, and range; statistical significance was determined by Wilcoxon’s rank sum test. For multiple comparisons, statistical significance was determined by the Kruskal-Wallis test followed by Holm’s step-down method. Wilcoxon’s signed rank test was used to assess differences in serum miR-99a-5p expression levels between patients before and after surgery. Differences were considered statistically significant at *p* < 0.05. A receiver operating characteristic (ROC) curve was generated and the area under the ROC curve (AUC) was calculated to evaluate the diagnostic accuracy of miR-99a-5p.

## Results

### Exosomes were collected from ovarian cancer cells and miR-99a-5p was found highly expressed in ovarian cancer-derived exosomes

**Fig. 1:**
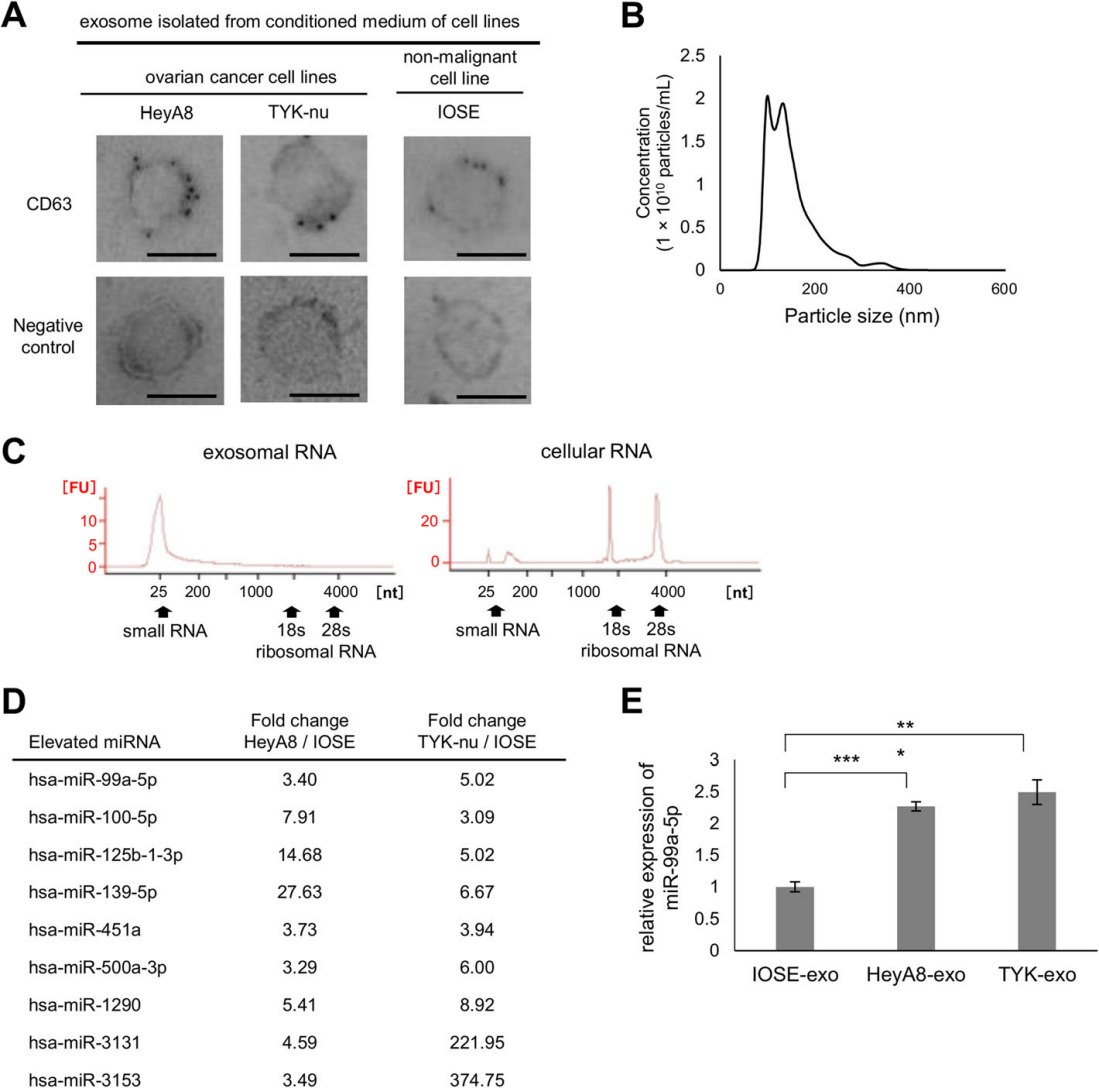
Exosomes were isolated from epithelial ovarian cancer (EOC) cell lines and then exosomal miRNAs were analyzed. **a** Electron microscopy. Exosomes were immunogold-labeled with anti-CD63 antibody. Transmission electron micrographs of purified exosomes secreted from several EOC cell lines are shown. Immortalized ovarian surface epithelium (IOSE) cells were used as a non-malignant control. Scale bar, 100 nm. Representative images are shown. **b** Nanoparticle tracking analysis. Concentration and size distribution of nano-sized particles in the exosome suspension were measured using a NanoSight system. The concentration presents the number of nano-sized particles per 1 mL culture medium. **c** RNA analyses. RNA was extracted from EOC-derived exosomes and analyzed using an Agilent 2100 Bioanalyzer (left). A profile of cellular RNA is shown as a control (right). Arrows indicate the position of small RNA and the 18S and 28S ribosomal RNAs. **d** Summary of exosomal miRNA microarray. Exosomal miRNAs that were upregulated by more than 3-fold in both EOC cells (HeyA8 and TYK-nu) vs. IOSE cells are listed. **e** miRNA qRT-PCR. Relative expression levels of miR-99a-5p in EOC-derived exosomes are shown. IOSE-derived exosomes were used as control. Data represent the mean ± standard deviation (SD) of three experiments. ***p < 0.001

First, we isolated EOC-derived exosomes from cell culture media using multi-step centrifugation. Two EOC cell lines, namely HeyA8 and TYK-nu, were used to isolate exosomes, as these cell lines are derived from high-grade serous carcinomas. An immortalized normal ovarian epithelial cell line, IOSE, was used as a control. Exosomes showed positive staining for CD63 (a representative exosome marker), exhibited a round-shaped morphology, and were approximately 100 nm in size as determined by electron microscopy (Fig. 1a) and nanoparticle analysis (Fig. 1b). To confirm whether exosomes were successfully purified, we analyzed the profile of total RNA extracted from exosomes using capillary electrophoresis. In contrast to total RNA extracted from cells, 18S and 28S ribosomal subunits were hardly detected in exosome sample extracts. Instead, the majority of total RNA in exosomes was below 2 kb, suggesting that most exosomal RNAs were small RNAs such as miRNAs, as previously reported [11] (Fig. 1c).

Next, to identify which exosomal miRNAs were highly expressed in ovarian cancer, miRNA microarrays were performed using exosomes from HeyA8, TYK-nu, and IOSE cells. Nine miRNAs were highly (> 2.0) expressed in both HeyA8 and TYK-nu cells in comparison with IOSE cells, as shown in Fig. 1d. Among those, we focused on miR-99a-5p, as miRNA qRT-PCR assays validated that its expression was significantly increased in EOC cell-derived exosomes; an increase of 3.4-fold in HeyA8 exosomes and 5.0-fold in TYK-nu exosomes compared with IOSE exosomes was observed (Fig. 1e).

### Serum miR-99a-5p is significantly elevated in ovarian cancer patients and reflects tumor burden

**Fig. 2:**
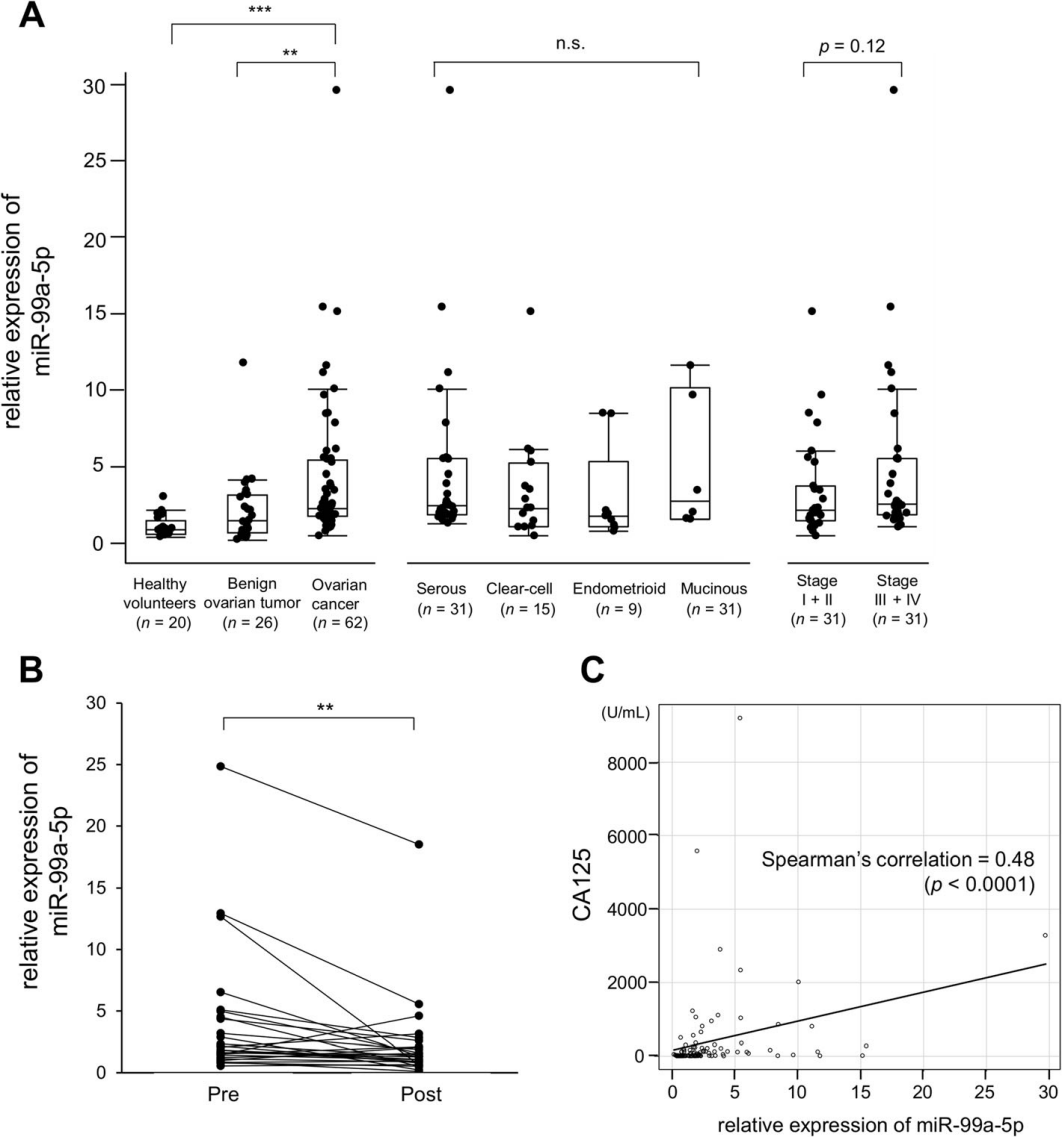
Serum miR-99a-5p expression is significantly upregulated in EOC patients. **a** Relative miR-99a-5p expression levels in sera from healthy volunteers (*n* = 20), patients with benign tumors (*n* = 26), and patients with EOC (*n* = 62) are shown (left). Relative serum miR-99a-5p expression levels among four different histological types (middle) and between EOC patients with early (I + II; *n* = 31) and late (III + IV) stage (*n* = 31; right) diseases are shown. The average miR-99a-5p expression levels of healthy volunteers (*n* = 20) were set to 1.0. The box-and-whisker plots indicate the median, interquartile range, and range. **p < 0.01 and ***p < 0.001. n.s., not significant. **b** Relative miR-99a-5p expression levels in matched serum samples from EOC patients before and after primary debulking surgery (PDS). Individual changes in serum expression levels of miR-99a-5p in EOC patients (*n* = 26) before (Pre) and after (Post) surgical removal of tumors are shown. The average miR-99a-5p expression levels of healthy volunteers (*n* = 20) was set to 1.0. **p < 0.01. ***p < 0.001. **c** Correlation analysis between expression levels of CA125 and miR-99a-5p. Spearman’s correlation analysis was performed to evaluate whether there was any association between the sera expression levels of miR-99a-5p and CA125

To evaluate the diagnostic value of miR-99a-5p as an EOC biomarker, miR-99a-5p expression levels were measured by qRT-PCR in sera from 62 EOC patients. Sera from 26 patients with benign ovarian tumors and 20 healthy volunteers were analyzed as controls. Patient characteristics are summarized in Table 1. In patients with EOC, serum miR-99a-5p levels were significantly higher than in healthy controls and patients with benign tumors (2.8-fold and 1.7-fold, respectively; Fig. 2a). EOC consists of the following four major histological subgroups: serous, clear-cell, endometrioid, and mucinous. However, among the histological subtypes, serum miR-99a-5p expression did not significantly differ (Fig. 2a). While the relative expression levels of miR-99a-5p in stage I-II EOC patients were 2.0 (1.4–3.6), expression levels in stage III-IV patients tended to increase to 2.5 (1.8–5.4, *p* = 0.12), indicating that miR-99a-5p reflects tumor burden. To further analyze this, sera were collected from 26 EOC patients before (2 or 3 days before PDS) and after surgery (36 ± 12 days after PDS) and then miR-99a-5p expression levels were compared (Fig. 2b). When the average miR-99a-5p expression levels in healthy volunteers (*n* = 20) was set to 1.0, relative expression levels before surgery were 1.8 (1.2–4.5) and significantly declined to 1.3 (0.8–2.1) after surgery (*p* = 0.003), strongly suggesting that serum miR-99a-5p reflects tumor burden and is derived from ovarian cancer cells. CA125, a conventional biomarker of EOC, is expressed as a membrane-bound protein at the surface of cells that undergo metaplastic differentiation into Müllerian-type epithelium or is released in soluble form in bodily fluids [12]. Thus, serum miR-99a-5p expression levels were compared with CA125 values and a positive correlation was observed in patients with EOC (*r* = 0.48, *p* < 0.0001; Fig. 2c).

**Table 1:** Participant characteristics

	Ovarian cancer		Benign ovarian tumor		Healthy volunteers
	(*n* = 62)		(*n* = 26)		(*n* = 20)
Age, years Median (range)	59 (19–78)		52 (16–81)		34.5 (24–45)
Histologic type	Serous	32 (52%)	Mucinous cystadenoma	9 (34%)	
	Clear	15 (24%)	Teratoma	7 (27%)	
	Endometrioid	9 (14%)	Endometrioma	4 (15%)	
	Mucinous	6 (10%)	Serous cystadenoma	2 (8%)	
			Thecoma	2 (8%)	
			Fibroma	1 (4%)	
			Fibrothecoma	1 (4%)	
FIGO Stage	I	26 (42%)			
	II	5 (8%)			
	III	24 (39%)			
	IV	7 (11%)

### Can serum miR-99a-5p potentially serve as an EOC biomarker?

**Fig. 3:**
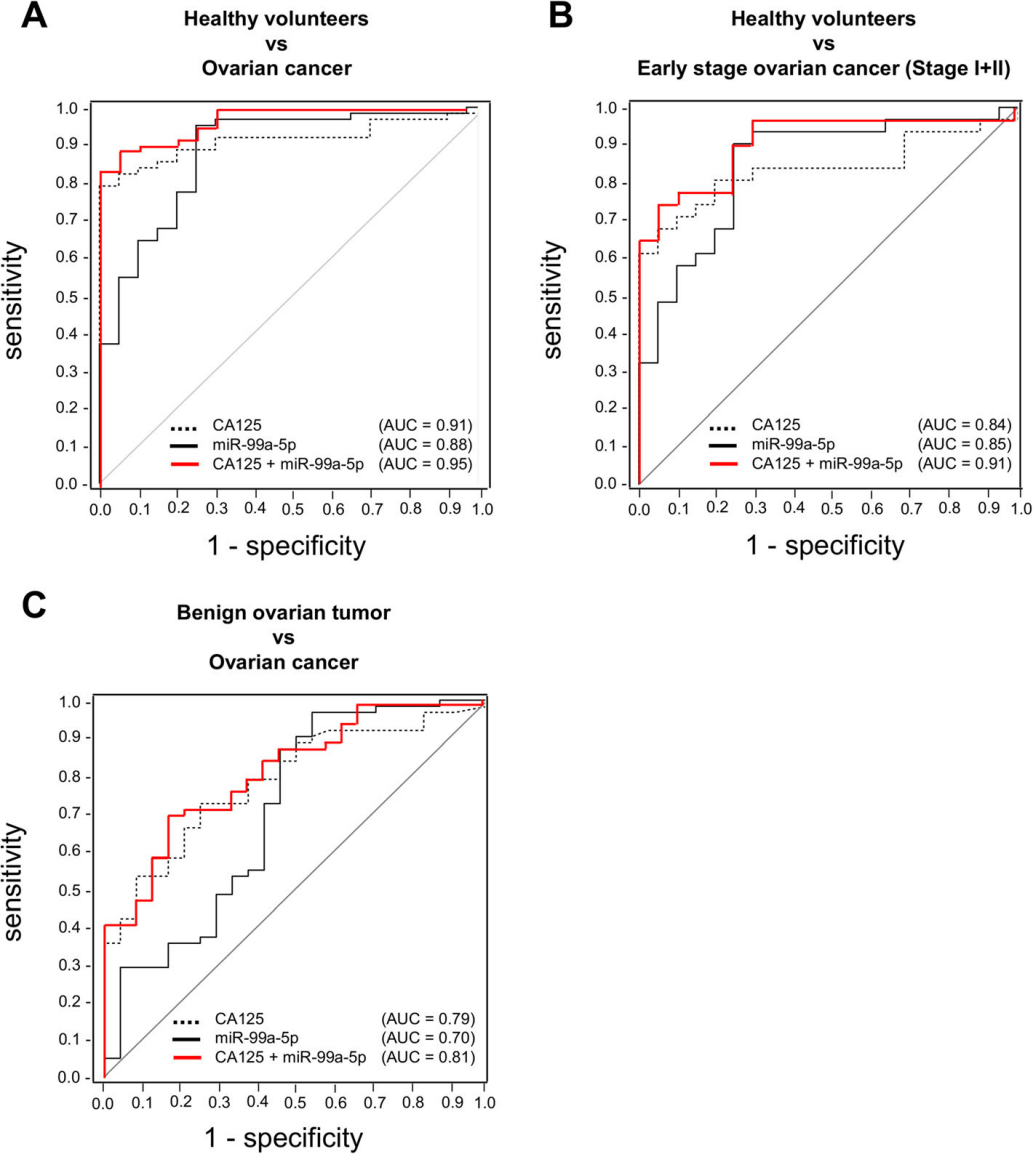
Diagnostic outcomes of serum miR-99a-5p for the prediction of EOC. **a** ROC analysis for miR-99a-5p (black line), CA125 (dotted line), and both miR-99a-5p and CA-125 together (red line) for the discrimination of patients with EOC (*n* = 62) from healthy volunteers (*n* = 20). **b** ROC analysis for miR-99a-5p (black line), CA125 (dotted line), and both miR-99a-5p and CA125 together (red line) for the discrimination of patients with early-stage EOC (*n* = 31) from healthy volunteers (*n* = 20). **c** ROC analysis for miR-99a-5p (black line), CA125 (dotted line), and both miR-99a-5p and CA125 together (red line) for the discrimination of patients with EOC (*n* = 62) from those with benign tumors (*n* = 26). Each AUC value is shown on the graph

To further evaluate the possibility of serum miR-99a-5p as a biomarker of EOC, ROC analysis for discriminating EOC from healthy volunteers was performed (Fig. 3a). When the cut-off for miR-99a-5p was set to 1.41, the sensitivity was 0.85 and the specificity was 0.75 (AUC = 0.88). For comparison with CA125, a ROC curve for discriminating EOC from healthy volunteers was similarly calculated. When the cut-off for CA125 was set to 35.0 U/mL, the sensitivity and specificity of serum CA125 were 0.82 and 0.95, respectively (AUC = 0.91). There was no significant difference between the AUCs of miR-99a-5p and CA125. When CA125 was combined with miR-99a-5p, the AUC improved from 0.91 to 0.95 (*p* = 0.09; Fig. 3a). Further statistical analyses were performed to develop classification models that can distinguish among different EOC subtypes. As shown in Additional file 1: Figure S1, the miR-99a-5p classification model had AUC, sensitivity, and specificity values of 0.60, 0.84, and 0.40, respectively, for serous EOC; 0.54, 0.33, and 0.91, respectively, for clear-cell EOC; 0.68, 0.67, and 0.82, respectively, for endometrioid EOC; and 0.56, 0.33, and 0.91, respectively, for mucinous EOC. Unfortunately, this model failed to distinguish between the different EOC subtypes.

Detecting EOC patients at an early stage to improve prognosis is clinically indispensable. For this reason, ROC analysis for discriminating early-stage (stage I-II) EOC from healthy volunteers was performed (Fig. 3b). When the cut-off of miR-99a-5p was set to 1.36, the sensitivity was 0.90 and the specificity was 0.75 (AUC = 0.85). The sensitivity and specificity of serum CA125 were 0.68 and 0.95, respectively (AUC = 0.84); Therefore, there was no significant difference between the AUCs of miR-99a-5p and CA125. When CA125 was combined with miR-99a-5p, the AUC improved from 0.84 to 0.91 (*p* = 0.13; Fig. 3b). Finding a noninvasive method to distinguish benign tumors from malignant cancers is critical as it is currently impossible to establish a definite diagnosis without surgery. For this reason, ROC analysis for discriminating EOC from benign tumors was performed (Fig. 3c). When the cut-off of miR-99a-5p was set to 1.36, the sensitivity was 0.87 and the specificity was 0.54 (AUC = 0.70). The sensitivity and specificity of serum CA125 were 0.73 and 0.75, respectively (AUC = 0.79) and thus, there was no significant difference between the AUCs of miR-99a-5p and CA125. When CA125 was combined with miR-99a-5p, the AUC improved from 0.79 to 0.81 (*p* = 0.51; Fig. 3c).

### Exosomal transfer of miR-99a-5p to HPMCs promotes EOC cell invasion

**Fig. 4:**
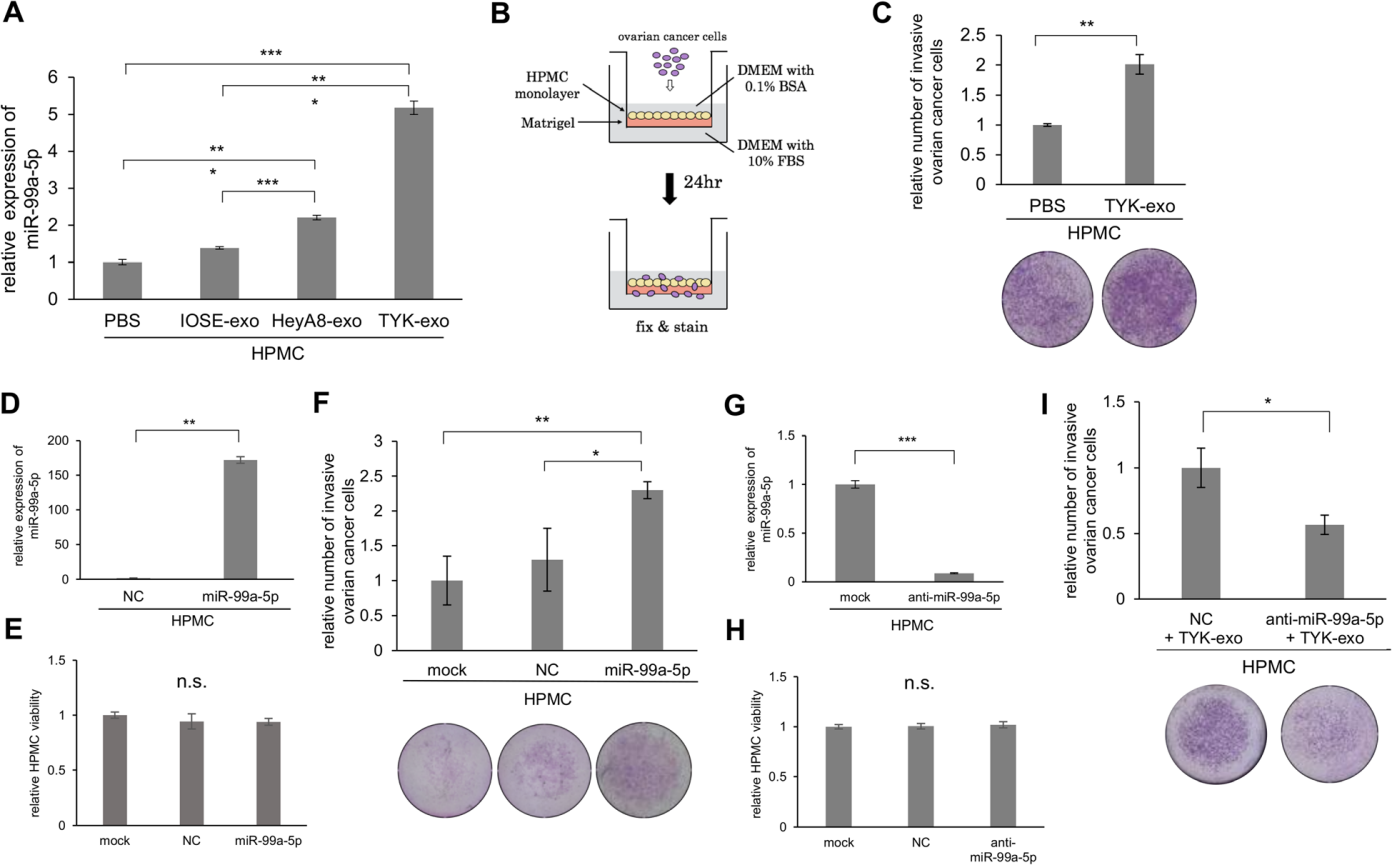
Exosomal transfer of miR-99a-5p to human peritoneal mesothelial cells (HPMCs) promotes EOC cell invasion. **a** miRNA qRT-PCR. Relative expression levels of miR-99a-5p in HPMCs treated with IOSE- and EOC-derived exosomes at 100 μg/mL for 24 h are shown. Expression levels of miR-99a-5p in HPMCs treated with PBS alone were set to 1.0. Data represent the mean ± SD of three experiments. **b** A schematic of the HPMC-coated in vitro invasion assay. HPMCs were plated onto 24-well cell culture inserts coated with Matrigel. After HPMCs reached confluence, cancer cells were incubated on the HPMCs monolayer and allowed to invade for 24 h. Cells that migrated to the trans-side of the membrane were quantitated. **c** HPMCs were pre-cultured with TYK-nu-derived exosomes at 100 μg/mL for 24 h and then TYK-nu cells were plated onto the HPMC monolayer. Twenty-four hours after incubation, invading cells on the underside of the filter were counted. Representative pictures of the chambers are shown below. **d** miRNA qRT-PCR. Relative expression levels of miR-99a-5p in HPMCs transfected with miR-99a-5p are shown. **e** In vitro cell proliferation assay. HPMCs were transfected with negative control miRNA or precursor miR-99a-5p. Twenty-four hours later, relative cell proliferation was assessed using the MTS assay. **f** HPMC-coated in vitro invasion assay. After HPMCs were transfected with negative control miRNA or precursor miR-99a-5p, TYK-nu cells were plated onto the HPMC monolayer and allowed to invade. **g** miRNA qRT-PCR. Relative expression levels of miR-99a-5p in HPMCs transfected with anti-miR-99a-5p are shown. **h** In vitro cell proliferation assay. HPMCs were transfected with negative control miRNA and anti-miR-99a-5p. **i** HPMC-coated in vitro invasion assay. After HPMCs transfected with negative control miRNA or precursor anti-miR-99a-5p were pre-cultured with TYK-exo, TYK-nu cells were plated onto the HPMC monolayer and allowed to invade. Data represent the mean ± SD of three experiments. *p < 0.05, **p < 0.01, and ***p < 0.001. n.s., not significant

Since exosomal miR-99a-5p is secreted from ovarian cancer cells and serum miR-99a-5p reflects tumor burden, we were encouraged to analyze how exosomal miR-99a-5p affects ovarian cancer progression. During peritoneal dissemination of ovarian cancer, cells detached from the primary site of origin attach to the peritoneal cavity, which is lined with a single layer of mesothelial cells. Therefore, the initial interaction of cancer cells with mesothelial cells is a key process of ovarian cancer progression and several recent reports have shown that EOC-derived exosomes mediate this process [13, 14]. Thus, we collected HPMCs from gynecological surgeries and treated them with EOC-derived exosomes. miR-99a-5p levels increased significantly in HPMCs treated with EOC (HeyA8 and TYK-nu)-derived exosomes compared with those treated with IOSE-derived exosomes (Fig. 4a). Since TYK-nu derived exosomes drastically increased expression levels of miR-99a-5p in HPMCs (5.2-fold), TYK-nu derived exosomes were used for subsequent experiments. The role of EOC-derived exosomes in ovarian cancer invasion was studied using an in vitro invasion assay. To mimic the mesothelial layer covering the omentum and other peritoneal organs, a monolayer of HPMCs was plated onto growth factor-reduced Matrigel (Fig. 4b). Pre-treatment of HPMCs with TYK-nu-derived exosomes at a concentration of 100 μg/mL significantly stimulated ovarian cancer cell invasions (Fig. 4c). To clarify the role of exosomal miR-99a-5p in ovarian cancer progression, HPMCs were transfected with miR-99a-5p (Fig. 4d) and an in vitro invasion assay was performed. While the enforced expression of miR-99a-5p did not affect the proliferation of HPMCs (Fig. 4e), it significantly promoted EOC cell invasion (Fig. 4f). Furthermore, miR-99a-5p expression in HPMCs was inhibited by transfection with miR-99a-5p specific antagonist (Fig. 4g). An in vitro invasion assay was subsequently performed and the inhibition of miR-99a-5p expression in HPMCs also did not affect the proliferation of HPMCs (Fig. 4h), but significantly attenuated EOC cell invasion (Fig. 4i). Collectively, these results show that the exosomal transfer of miR-99a-5p into HPMCs promotes EOC cell invasion without affecting cell viability.

### EOC-derived exosomes enhance the expression of fibronectin and vitronectin in HPMCs thorough miR-99a-5p transfer

**Fig. 5:**
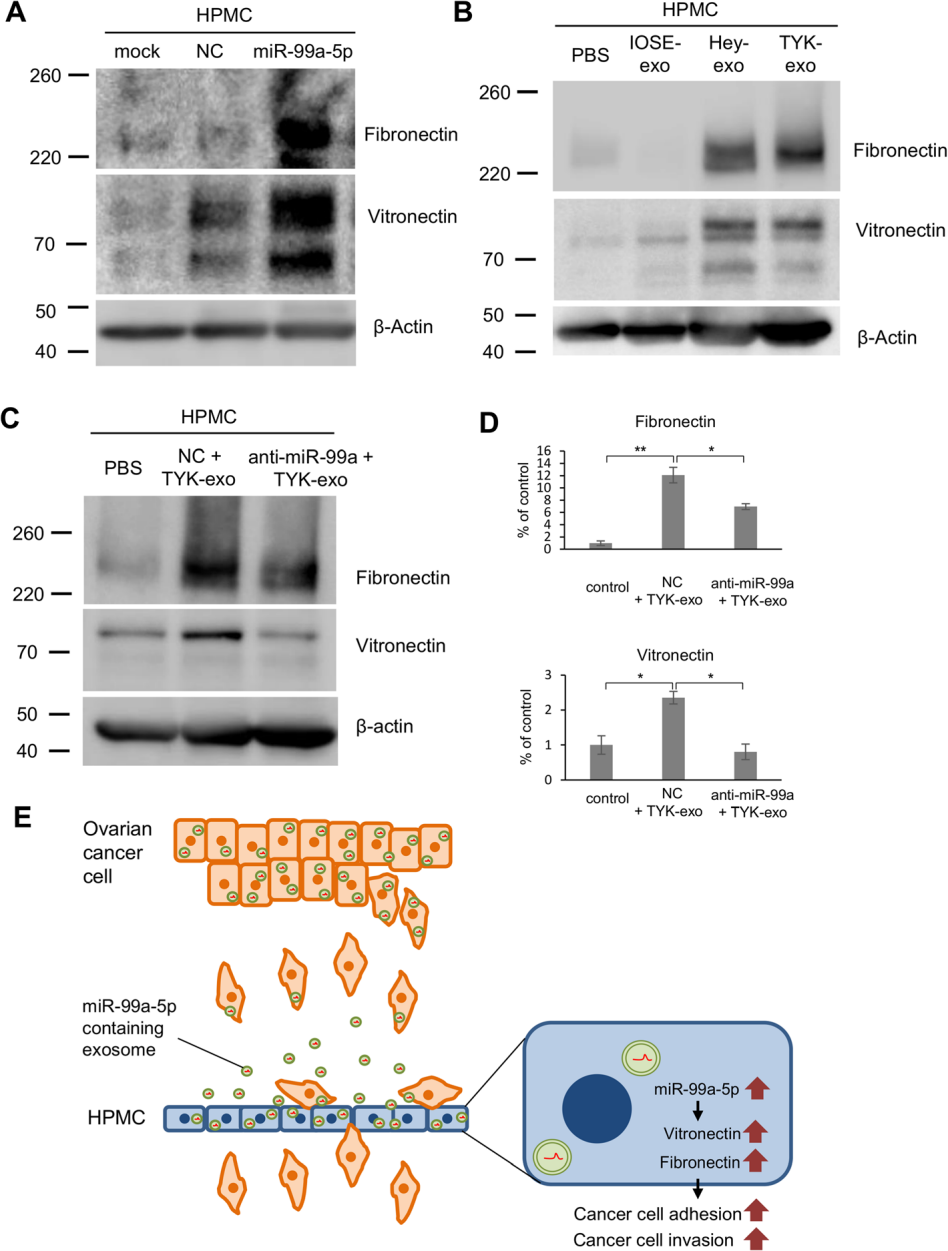
EOC-derived exosomes enhanced fibronectin and vitronectin expression in HPMCs through the transfer of miR-99a-5p. **a** Western blotting. HPMCs were transfected with miR-99a-5p or negative control miRNA. Thereafter, cell lysates were collected, and immunoblotting was performed with antibodies against fibronectin, vitronectin, or β-actin as a loading control. Representative blots from three independent experiments are shown. **b** After HPMCs were treated with IOSE- and EOC-derived exosomes at 100 μg/mL for 24 h, cell lysates were collected, and western blotting was performed as described above. **c** HPMCs transfected with negative control miRNA and anti-miR-99a-5p were treated with exosomes at 100 μg/mL for 24 h. Thereafter, cell lysates were collected, and western blotting was performed as described above. **d** Densitometric ratios of fibronectin, vitronectin, and β-actin expression. **e** Model. EOC-derived exosomes affect HPMCs through the transfer of miR-99a-5p. See text for details

To gain insights into the mechanism through which exosomal miR-99a-5p promotes EOC cell invasion, comprehensive proteomics analyses were performed through the TMT method. In HPMCs transfected with miR-99a-5p, several proteins were upregulated (more than 1.5-fold) compared with those transfected with negative control miRNA (Table 2). Since the enforced expression of miR-99a-5p in HPMCs promoted EOC cell invasion, we focused on fibronectin and vitronectin, which are related to extracellular matrix organization. As a validation assay, western blotting was performed and HPMCs transfected with miR-99a-5p showed significantly enhanced expression of fibronectin and vitronectin compared with that in cells transfected with negative control (Fig. 5a). Similarly, transfection of HPMCs with EOC-derived exosomes, compared with IOSE-derived exosomes, drastically enhanced the expression of fibronectin and vitronectin (Fig. 5b). The enhanced expression of these proteins was significantly attenuated by transfection with the specific inhibitor of miR-99a-5p (Fig. 5c, d), suggesting that EOC-derived exosomes upregulate the expression of fibronectin and vitronectin in HPMCs through the transfer of miR-99a-5p.

**Table 2:** Quantitative proteomics by the Tandem Mass Tag (TMT) method

Functional grouping	Protein	Fold change (miR-99a-5p/NC)
Regulation of catalytic activity	Superoxide dismutase	2.32
	Serine/threonine-protein phosphatase	2.23
Antigen processing and presentation of peptide antigen via MHC class I	Antigen peptide transporter 1	2.01
	GTP-binding protein SAR1b	1.70
Mitochondrion organization	Stomatin-like protein 2	1.87
	Single-stranded DNA-binding protein	1.70
Cell-cell adhesion	60S ribosomal protein L29	1.85
	60S ribosomal protein L15	1.72
	Acyl-protein thioesterase 2	1.62
ER to Golgi vesicle-mediated transport	Dynactin subunit 1	1.72
	Protein transport protein Sec23B	1.66
rRNA processing	40S ribosomal protein S27	1.64
Translation	40S ribosomal protein S26	1.60
Translational initiation		
Negative regulation of blood coagulation	PAI-1	1.62
Extracellular matrix organization	Vitronectin	1.53
	Fibronectin	1.52
Other	Dipeptidyl peptidase 4	2.03
	Thioredoxin-like protein 1	1.88
	THO complex subunit 4	1.79
	Synaptosomal-associated protein 23	1.66
	Tetraspanin	1.64
	Cofilin-2	1.63

Fold-changes in expression levels of each protein between HPMCs transfected with miR-99a-5p and those transfected with negative control miRNA are listed. Proteins were categorized by gene ontology (GO) based on biological process (BP) using DAVID 6.8 Functional Annotation Bioinformatics Analysis

## Discussion

During the past decade, exosomes have been shown to play important roles in cancer progression through the intercellular transfer of proteins, mRNA, and miRNA [15]. Unique circulating exosomal miRNA signatures have been recently identified in several cancers, seeing miRNAs are secreted from various cells, including cancer cells, into body fluids via exosomes [16]. Therefore, exosomal miRNAs in body fluids may be useful diagnostic biomarkers for the detection of cancer [8]. Herein, we identified that miR-99a-5p is upregulated in exosomes derived from EOC cells and highly expressed in sera from EOC patients. Furthermore, we revealed that exosomal miR-99a-5p derived from EOC cells promotes cell invasion by affecting HPMCs at least in partly through fibronectin and vitronectin upregulation. We provided novel insights into how this exosomal miRNA modulates the peritoneal dissemination of ovarian cancer as summarized in Fig. 5e.

The early detection of EOC is indispensable for improving patient outcomes, considering that patients diagnosed with early-stage EOC have favorable prognoses and that optimal prevention of EOC occurrence is not realistically practical. In addition to CA125, human epididymal secretory protein 4 (HE4) has emerged as a new biomarker for ovarian cancer. For instance, Wei et al. [17] compared the expression levels of serum CA125 and HE4 in 158 individuals, including 64 patients with ovarian cancer, 64 with ovarian benign tumors, and 30 healthy individuals, and reported high sensitivity and specificity of HE4 (75% and 98%, respectively) for the diagnosis of ovarian cancer. Thus, the combined measurement of CA125 together with HE4 may facilitate the early diagnosis of ovarian cancer and improve the evaluation of disease status although the evidence is still lacking [18]. Li et al. [19] stated in their comprehensive review of 11 studies that HE4 is not better than CA125 for the prediction of EOC. Thus, improved biomarkers for EOC detection remain to be identified and one candidate is circulating miRNAs, as they exist stably in circulating blood, reflect tissue or organ conditions, and are present in circulating microvesicles such as exosomes.

Since 2008, various studies have demonstrated the clinical relevance of circulating miRNAs as diagnostic and prognostic biomarkers for ovarian cancer, using blood plasma or serum as reviewed previously by us [7]. Taylor et al. [20] first reported that eight exosomal miRNAs (miR-21, miR-141, miR-200a, miR-200b, miR-200c, miR-203, miR-205, and miR-214) from sera were elevated in ovarian cancer patients compared benign controls. They reported that the miRNA signatures from exosomes were parallel to those from the originating tumor cells, indicating that circulating miRNA profiles accurately reflect tumor profiles [20]. In a cohort of 163 EOC patients, the levels of miR-200b and miR-200c were higher in stage III-IV patients than in stage I-II patients [21]. Recently, Yokoi et al. [22] developed a novel predictive model using a combination of eight circulating serum miRNAs (miR-200a-3p, miR-766-3p, miR-26a-5p, miR-142-3p, let-7d-5p, miR-328-3p, miR-130b-3p, and miR-374a-5p) that was able to distinguish ovarian cancer patients from healthy controls with high sensitivity and specificity (0.92 and 0.91, respectively) [22]. They also demonstrated that most of these eight miRNAs were packaged in extracellular vesicles, including exosomes, and were derived from ovarian cancer cells. In the present study, we first identified that serum miR-99a-5p expression levels were specifically elevated in EOC patients. Furthermore, serum miR-99a-5p expression levels decreased after PDS, indicating that serum miR-99a-5p reflects tumor burden. However, conflicting results have been reported regarding the potential of miR-99a-5p as a cancer biomarker [23–26]. Indeed, miR-99a expression often decreases in cases with prostate, bladder, or breast cancers [25, 26]. Further elucidation with a larger cohort is needed to confirm our findings.

Interactions occurring between cancer cells at the invasive front and cells in the tumor microenvironment promote cancer invasion and metastasis [27]. Recent reports have revealed that exosomes mediate this cell-cell communication by carrying a variety of proteins, mRNAs, or miRNAs and can significantly influence the phenotype of recipient cells [28]. In peritoneal dissemination of ovarian cancer, exosomes released from cancer cells promote ovarian cancer progression by affecting neighboring HPMCs [13, 14]. Nakamura et al. [13] reported that EOC-derived exosomes transfer CD44 to HPMCs. Upon exosome uptake, HPMCs underwent a change in cellular morphology to a mesenchymal, spindle phenotype, facilitating cancer invasion. Yokoi et al. [14] reported that EOC-derived exosomes carry *MMP1* mRNA to HPMCs, inducing apoptosis and facilitating peritoneal dissemination. Exosomal miRNAs have been implicated in a variety of processes such as oncogenesis, drug resistance, cancer invasion, and metastasis [28, 29]. In ovarian cancer, exosomes derived from stromal cells deliver miR-21 to ovarian cancer cells, leading to chemo-resistance by targeting APAF1 [30]. To our knowledge, this study is the first to reveal that exosomal miR-99a-5p is transferred from EOC cells to HPMCs, facilitating EOC cell invasion. Conflicting results have been reported regarding the function of miR-99a in cancer biology. In acute myeloid leukemia cells, the ectopic expression of miR-99a resulted in increased colony formation, cell proliferation, and chemoresistance by upregulating genes associated with stem cell maintenance, cell cycle, and MYC target genes [23]. On the contrary, miR-99a overexpression in breast cancer cells reduced cell viability and cell apoptosis by targeting the mammalian target of rapamycin (mTOR) [31]. Turcatel et al. [32] reported that miR-99a modulated the transforming growth factor (TGF)-β-induced epithelial to mesenchymal transition by inhibiting SMAD3 phosphorylation and that overexpression of miR-99a increased fibronectin expression in normal murine mammary gland cells, which was consistent with our data.

In the present study, we showed that exosomal miR-99a-5p derived from EOC cells increased the expression of fibronectin and vitronectin in neighboring HPMCs. Numerous previous studies have revealed that both fibronectin and vitronectin play important roles in peritoneal dissemination of ovarian cancer [33, 34]. Kenny et al. [33] showed that the knockdown of fibronectin impaired EOC cell adhesion, invasion, and proliferation, which led to the inhibition of metastases in vivo. Heyman et al. [34] showed that vitronectin contributes to the ability of EOC cells to bind to mesothelial cells. Thus, the increased expression of these extracellular matrix proteins induced by exosomal miR-99a-5p may contribute to EOC cell invasion.

Several limitations of this study should be acknowledged. In the present study, the potential advantage of combining multiple biomarkers vs. the use of single biomarkers alone was shown. However, due to a small sample size, we were unable to fully investigate other factors as would be required to develop a multi-biomarker panel. In particular, we failed to show differences among histological subtypes of EOC in our cohort, although Yokoi et al. [22] indicated that the expression levels of circulating serum miRNAs can be representative of histological subtypes. Further research with a larger cohort is required to pursue the potential of serum miR-99a-5p as an EOC biomarker. We also failed to show the direct target of miR-99a-5p to elucidate how exosomal miR-99a-5p modulates the expression of fibronectin and vitronectin. More detailed studies are needed to clarify the exact mechanism.

## Conclusion

We demonstrated that exosomal miR-99a-5p is elevated in the sera of ovarian cancer patients and targeting this exosomal miRNA may serve as a target for inhibiting ovarian cancer progression. Although many challenges would need to be overcome before the large-scale clinical utilization of miR-99a-5p in ovarian cancer treatment, its potential is exciting deserves further investigation.

## Additional file

Additional file 1: **Figure S1.** ROC analysis discriminating specific EOC histologic types from other subtypes. (PPTX 143 kb)

## Abbreviations

AUC: Area under the receiver operator characteristic curve
CA125: Carbohydrate antigen 125
EOC: Epithelial ovarian cancer
FIGO: The International Federation of Gynecologists and Obstetricians
HE4: Human epididymal secretory protein 4
HPMC: Human peritoneal mesothelial cell
miRNA: microRNA
ROC: Receiver operator characteristic
TMT: Tandem mass tag
