# Supplementary material for: Exosomal miR-99a-5p is elevated in sera of ovarian cancer patients and promotes cancer cell invasion by increasing fibronectin and vitronectin expression in neighboring peritoneal mesothelial cells
Source: BMC Cancer. 2018 Nov 5;18:1065. doi: 10.1186/s12885-018-4974-5 (PMC6217763; doi:10.1186/s12885-018-4974-5)
Supplement: Supplementary file 1 — Figure S1. ROC analysis discriminating specific EOC histologic types from other subtypes. (PPTX 143 kb) [file 12885_2018_4974_MOESM1_ESM.pptx]

## Slide 1
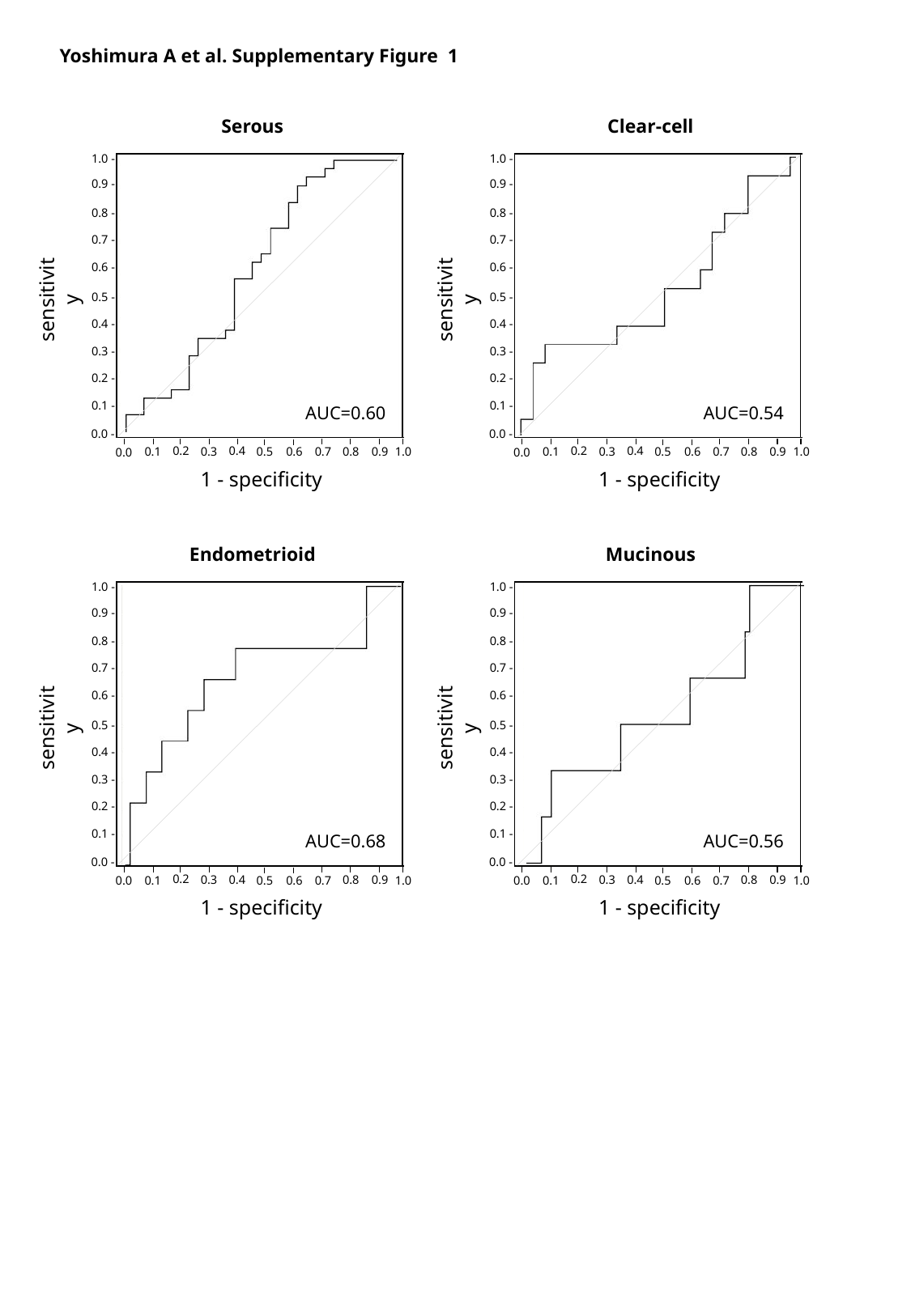

Yoshimura A et al. Supplementary Figure 1
Serous
Clear-cell
1.0 -
1.0 -
0.9 -
0.9 -
0.8 -
0.8 -
0.7 -
0.7 -
0.6 -
0.6 -
sensitivity
sensitivity
0.5 -
0.5 -
0.4 -
0.4 -
0.3 -
0.3 -
0.2 -
0.2 -
0.1 -
0.1 -
AUC=0.60
AUC=0.54
0.0 -
0.0 -
0.2
0.2
0.4
0.4
0.9
0.9
0.3
0.8
0.3
0.8
0.5
0.5
0.1
0.6
0.7
1.0
0.1
0.6
0.7
1.0
0.0
0.0
1 - specificity
1 - specificity
Endometrioid
Mucinous
1.0 -
1.0 -
0.9 -
0.9 -
0.8 -
0.8 -
0.7 -
0.7 -
0.6 -
0.6 -
sensitivity
sensitivity
0.5 -
0.5 -
0.4 -
0.4 -
0.3 -
0.3 -
0.2 -
0.2 -
0.1 -
0.1 -
AUC=0.68
AUC=0.56
0.0 -
0.0 -
0.2
0.2
0.4
0.4
0.9
0.9
0.3
0.8
0.3
0.8
0.5
0.5
0.1
0.6
0.7
1.0
0.1
0.6
0.7
1.0
0.0
0.0
1 - specificity
1 - specificity
